# CYP2E1 Rsa Ι/Pst Ι polymorphism and lung cancer susceptibility: A meta‐analysis involving 10,947 subjects

**DOI:** 10.1111/jcmm.13668

**Published:** 2018-05-29

**Authors:** Ze‐Tian Shen, Xin‐Hu Wu, Bing Li, Jun‐shu Shen, Zhen Wang, Jing Li, Xi‐Xu Zhu

**Affiliations:** ^1^ Department of Radiation Oncology Jinling Hospital Medical School of Nanjing University Nanjing China

## Abstract

The above article, published in the *Journal of Cellular and Molecular Medicine* on 14 September 2016 in Wiley Online Library (wileyonlinelibrary.com), and in Volume 19, pp. 2136‐2142, has been retracted by agreement between the authors, the journal Editor in Chief, Stefan Constantinescu, and John Wiley & Sons Ltd. The retraction has been agreed due to unattributed overlap of the language used in the “Materials and method” and “Discussion” sections of this study and the following article published in *Lung Cancer*: “CYP2E1 Rsa I/Pst I polymorphism is associated with lung cancer risk among Asians” by Ping Zhan, Jing Wang, Yu Zhang, Li‐Xin Qiu, Su‐feng Zhao, Qian Qian, Shu‐Zhen Wei, Li‐Ke Yu and Yong Song, Volume 69, 2010, pages 19‐25.

**REFERENCE**

Shen Z‐T, Wu X‐H, Li B, Shen J‐S, Wang Z, Li J, Zhu X‐X. CYP2E1 Rsa Ι/Pst Ι polymorphism and lung cancer susceptibility: a meta‐analysis involving 10,947 subjects. J Cell Mol Med. 2015;19:2136‐2142. https://doi.org/10.1111/jcmm.12579

The above article, published in the *Journal of Cellular and Molecular Medicine* on 14 September 2016 in Wiley Online Library (wileyonlinelibrary.com), and in Volume 19, pp. 2136‐2142, has been retracted by agreement between the authors, the journal Editor in Chief, Stefan Constantinescu, and John Wiley & Sons Ltd. The retraction has been agreed due to unattributed overlap of the language used in the “Materials and method” and “Discussion” sections of this study and the following article published in *Lung Cancer*: “CYP2E1 Rsa I/Pst I polymorphism is associated with lung cancer risk among Asians” by Ping Zhan, Jing Wang, Yu Zhang, Li‐Xin Qiu, Su‐feng Zhao, Qian Qian, Shu‐Zhen Wei, Li‐Ke Yu and Yong Song, Volume 69, 2010, pages 19‐25.

**REFERENCE**

Shen Z‐T, Wu X‐H, Li B, Shen J‐S, Wang Z, Li J, Zhu X‐X. CYP2E1 Rsa Ι/Pst Ι polymorphism and lung cancer susceptibility: a meta‐analysis involving 10,947 subjects. J Cell Mol Med. 2015;19:2136‐2142. https://doi.org/10.1111/jcmm.12579

